# Contrastive learning-driven spatiotemporal dynamically adaptive framework for stylized 3D human motion generation

**DOI:** 10.1371/journal.pone.0337596

**Published:** 2026-02-19

**Authors:** Zhiqiang Song, Ruyan Zhang, Shuangjun Li, Chao Liu

**Affiliations:** 1 College of Physical Education, Shandong Sport University, Jinan, China; 2 Faculty of Health Sciences and Sports, Macao Polytechnic University, Macao, China; 3 Physical Education Institute, Yanching Institute of Technology, Langfang, China; FEI: Centro Universitario da FEI, BRAZIL

## Abstract

Most existing methods for 3D human motion generation focus primarily on global style statistics in the temporal dimension, which limits their ability to capture local stylistic variations in dynamic motions. This often results in generated sequences lacking expressive detail. To address this challenge, a contrastive learning-driven framework is proposed for spatiotemporal dynamically adaptive stylized 3D human motion generation. Building upon conventional spatial attention (SA) and temporal attention (TA) modules, two instance normalization variants—spatial attention instance normalization (SAIN) and temporal attention instance normalization (TAIN)—are introduced to disentangle and extract motion style features from local and global perspectives, respectively. Simultaneously, a dual-path structure is employed to isolate pure motion content at both local and global levels, ensuring effective separation of style and content information. A style injector, composed of spatially adaptive dynamic attention (SADA) and temporally adaptive dynamic attention (TADA) modules, is developed to integrate the extracted style features with motion content in a temporally and spatially ordered manner, enabling fine-grained style injection. During training, style contrastive loss and content contrastive loss are incorporated to enforce compact clustering of features with similar styles or contents in the feature space, while promoting separation of dissimilar ones. This enhances both the stylistic diversity and content fidelity of the generated sequences. Comprehensive experiments conducted on the Xia dataset demonstrate the superior performance of the proposed method, achieving an FID of 0.06, accuracy of 96.70%, diversity of 5.67, and multimodality of 0.97, all of which are close to real data (FID 0.01). In the motion style transfer task, our model attains 94.11 CRA and 89.41 SRA, outperforming state-of-the-art baselines.

## 1. Introduction

Motion generation based on human motion capture technology primarily involves recording joint positions and body movements using motion capture devices, converting real human motion into digital data [1]. These techniques have been widely employed across entertainment industries such as film, television, and video games, as well as in motion analysis, medical rehabilitation, and sports training [2]. However, due to the high cost associated with motion capture systems, there is a growing demand for technologies capable of automatically generating realistic human motion data to reduce production expenses. This demand has given rise to the task of human motion generation [3].

Human motion generation can generally be categorized into two types: unconditional generation, which synthesizes random motion sequences in space [4], and conditional generation, which produces motions based on specific inputs such as music [5] or predefined actions [6]. With advances in 3D motion synthesis, semantic action labels have increasingly been utilized as conditions for motion generation, enabling applications in script visualization [7], virtual animation [8], and robotic task planning [9]. While semantic-guided synthesis often results in high-quality motion, it remains challenged by the inherent diversity of motion styles.

**Fig 1:**
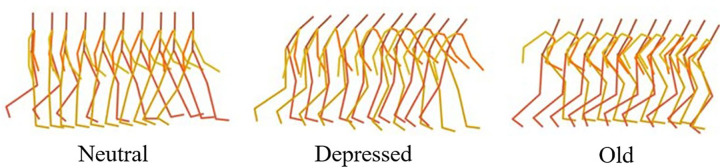
Visualization of human walking under different motion styles.

Motion style plays a critical role in character animation, as it reflects personality, emotion, age, and other nuanced human attributes (see Fig 1). The expressive representation of motion style is essential for rendering realistic and compelling virtual characters [10]. With the growing adoption of virtual characters in computer graphics and virtual reality, achieving fine-grained control over motion styles has become a significant challenge in character animation. Given the subjective and subtle nature of motion styles, capturing them typically requires professional actors or animators [11]. Moreover, the same motion style can be conveyed in multiple ways, making manual methods costly and time-consuming. Recent studies have explored extracting style features from reference motions and transferring them to other sequences to produce stylized results. While promising, these methods typically focus on global style statistics in the temporal domain and often overlook local variations [12]. As a result, they struggle to faithfully convey styles embedded in dynamic motions such as jumping. Furthermore, although existing style-aware 3D motion generation methods can produce smooth and stylized motion sequences, they often fail to capture spatial relationships between joints. This limitation hampers style transfer across action categories—for instance, when transferring from “proud-punching” to “elderly-jumping,” the outcome may still exhibit a proud style, but the expressiveness becomes attenuated due to the skeletal configuration during jumping. Additionally, the lack of constraints on the latent style space often leads to disorganized distributions, causing inconsistency and instability in the quality of the generated motions [13,14].

To enhance the quality and expressiveness of stylized 3D motion sequences, a contrastive learning-driven spatiotemporal dynamically adaptive generation framework is proposed in this study. Extensive experiments on public datasets validate the effectiveness of the proposed method. The main contributions are summarized as follows:

- A novel style disentanglement mechanism is introduced by extending conventional spatial attention (SA) and temporal attention (TA) modules into spatial attention instance normalization (SAIN) and temporal attention instance normalization (TAIN), enabling fine-grained extraction of motion content features at both local and global levels while mitigating style interference. This significantly improves both style modeling precision and motion content representation.
- A pair of dynamically adaptive modules—spatially adaptive dynamic attention (SADA) and temporally adaptive dynamic attention (TADA)—are proposed to modulate the intensity of style injection based on local spatiotemporal context, achieving more natural, coherent, and high-fidelity style transitions.
- During training, style and content contrastive losses are introduced to encourage clustering of features with similar style or content and separation of dissimilar ones within the latent space. This enhances the discriminative capacity of both the style and content extractors, thereby improving the diversity and stylistic expressiveness of the generated motion sequences.

The remainder of this paper is organized as follows: Section 2 presents review related work, Section 3 details the proposed framework, Section 4 presents the experimental setup, Section 5 discusses results, and Section 6 concludes with key findings and future directions.

## 2. Preliminary and related works

### 2.1. Preliminary

The core objective of human pose estimation and 3D human generation is to recover the spatial structure of the human body—such as joint coordinates or mesh vertices—and/or shape parameters (e.g., SMPL parameters) from a single image, multi-frame video, or depth input. The recovered results are expected to be geometrically accurate, semantically consistent with the input, and temporally coherent with smooth motion in video sequences. Common tasks include 2D keypoint estimation, 3D joint position estimation, parametric human reconstruction (e.g., SMPL regression), and fine-grained mesh reconstruction. Widely used evaluation metrics comprise MPJPE/PA-MPJPE, PCK, mesh reconstruction error, and reprojection error; in motion generation or sequence recovery scenarios, additional measures such as FID, diversity, motion smoothness (velocity/acceleration stability), and downstream utility (e.g., action recognition accuracy) are also employed. Recent advances can be categorized into several major technical directions, each with relevance to the present work: (1) Parametric regression enables end-to-end estimation of parameters such as SMPL, providing compact representations suitable for downstream applications. However, such parameterized forms are limited in recovering fine details and expressive styles. (2) Dense pixel-to-surface mapping with reprojection constraints employs IUV mappings and differentiable rendering to impose image-space supervision, improving geometric and appearance consistency. Yet, these methods are sensitive to occlusion and complex backgrounds and are computationally demanding. (3) Graph- and mesh-based neural networks with multi-resolution optimization model skeletal or mesh topology to improve local geometric and pose accuracy, but global semantic and shape consistency still heavily depend on input data. (4) Optimization-based approaches exploit priors and mutual constraints to achieve stable estimation under weak supervision, though temporal coherence and real-time performance remain challenging. (5) Temporal modeling focuses on video or motion sequences, leveraging sequence models to ensure smoothness and rhythm consistency. In particular, Transformer architectures excel at capturing long-range dependencies, providing a methodological basis for preserving temporal style coherence during generation. Together, these developments highlight two challenges that are central to this study and serve as key design motivations. Enhancing style expressiveness while preserving semantic fidelity of motion (i.e., retaining content-related accuracy while improving style-related accuracy). Jointly modeling local spatial structure and global temporal rhythm to achieve stylized motion that is both distinguishable and natural.

### 2.2. 3D human generation based on deep learning

With the advancement of deep learning, many researchers have begun to estimate human shape and pose directly from images [15] or videos [16] in an end-to-end manner. HMR [17] employs the SMPL parametric 3D human model and incorporates the idea of Generative Adversarial Networks (GANs) [18]. A discriminator is integrated into the loss function to evaluate the plausibility of generated parameters, while the reprojection error of body joints is added as an additional constraint. This approach enables direct regression of SMPL parameters from a single image, producing parameterized human representations that are efficient for downstream tasks such as animation and synthesis. Training and inference are relatively efficient; however, modeling capacity remains limited by the representational power of SMPL and the distribution of training data, leading to insufficient recovery of fine-grained details and surface textures. Creswell et al. [19] introduced DensePose, a deep learning framework that maps 2D pose and surface texture information from a single image to a 3D human surface. By performing pose estimation and dense surface alignment simultaneously, it delivers high-quality 3D reconstructions with pixel-level UV mapping, facilitating high-fidelity mesh and texture recovery. Despite its strength in detail modeling, DensePose is sensitive to occlusion and cluttered backgrounds, and the stability of full mesh recovery from IUV mappings depends on effective downstream reconstruction strategies. Xu et al. [15] advanced this line of work by incorporating mesh vertex reprojection errors into the loss function. Their method takes IUV maps, obtained from DensePose, as inputs and regresses body meshes, while a Differential Renderer (DR) is used to synthesize IUV images. The alignment error between predicted and input IUV maps is then minimized. By combining IUV mapping with differentiable rendering, this approach enforces pixel-level consistency between images and meshes, thereby enhancing geometric accuracy. Nonetheless, it requires high-quality IUV inputs and incurs significant computational cost, while still struggling under challenging lighting and occlusion conditions. Other works have sought to improve mesh reconstruction by jointly leveraging 2D and 3D information. For example, Lassner et al. [20] imposed mutual constraints between 2D and 3D poses to refine both pose and shape parameters, enabling robust reconstruction from monocular images, multi-view images, and depth data. However, the method depends heavily on well-designed loss functions and weights, as inconsistent supervision signals may hinder convergence. The development of graph convolutional networks (GCNs) has also spurred advances in 3D human generation. Kolotours et al. [21] proposed a coarse-to-fine mesh refinement architecture that explicitly models mesh topology using GCNs. While their method achieves accurate pose estimation, discrepancies remain between the reconstructed body shape and the true subject. Similarly, SMPLify [22] estimates human pose and shape from a single 2D image without requiring annotated 3D supervision. Yet, the method depends on an initial pose estimate, making it sensitive to initialization errors, and the resulting motion sequences often lack temporal smoothness. To address this issue, Liu et al. [23] proposed a Transformer-based framework for reconstructing 3D human meshes from monocular RGB videos. Compared with convolutional neural networks, the Transformer encoder provides superior modeling of temporal dependencies in human motion, leading to smoother reconstructions. Nevertheless, its performance gains are constrained by high computational and data requirements, with limited improvement in short sequences or low frame-rate settings.

### 2.3. Motion style transfer

The rapid progress of deep learning in computer vision and image generation has driven the emergence of a wide range of neural network-based methods for style transfer. Xia et al. [24] provided a large annotated motion dataset that separates motion style from motion content, modeling their differences through a mixture of autoregressive models. This dataset, together with the explicit proposal of style–content disentanglement, established a conceptual and data foundation for stylized motion generation. However, the dataset size and style coverage constrain generalization, and early methods required retraining or paired data to adapt to unseen styles. Holden et al. [25] leveraged large-scale motion capture data to train a framework that maps high-level control parameters to motion manifolds, enabling operations such as motion style transfer. Their approach utilized autoencoders and Gram matrix computations over motion sequence frames to edit style. While effective, this method relied heavily on paired or diverse data, making it less adaptive to rare or novel styles. The contributions of Xia and Holden thus laid the groundwork for subsequent research, yet the dependence on paired data made adaptation to new motion styles time-consuming and inefficient. To address temporal dependencies more explicitly, Wang et al. [26] modeled motion sequences using spatiotemporal recurrent neural networks. By partitioning skeletal joints and employing a spatial encoder, their method captured local spatiotemporal relations and simulated spatial variations. This architecture was well suited for sequence modeling but limited by recurrent networks’ difficulties in capturing long-range dependencies, particularly compared with Transformer-based approaches. Aberman et al. [27] introduced a data-driven style transfer method that eliminates the need for paired datasets by adopting Adaptive Instance Normalization (AdaIN) [28] to guide style transfer. This significantly reduced dependence on labeled pairs and enhanced scalability. Nevertheless, because AdaIN relies on statistical alignment, its ability to capture fine-grained temporal or structural variations remains limited, and careful design is required for skeletal data with strict continuity constraints. Building on this idea, Park et al. [29] combined AdaIN with Spatial-Temporal Graph Convolutional Networks (ST-GCNs) [30] to better capture temporal and spatial dynamics of skeletal motion, thereby producing higher-quality stylized sequences. However, integrating AdaIN with graph structures demands careful balancing, and complex styles may still result in blurred or unstable stylization. Kothari et al. [31] expanded the definition of motion style to include social norms governing how individuals move within social contexts. They proposed the Motion Style Adapter (MoSA) to predict motions in accordance with these norms. By embedding broader social attributes into style, this approach enhances the realism of motion in crowd or interaction scenarios. Yet, modeling such social norms remains constrained by the availability of contextual data and labels, and generalization across cultures or scenarios requires additional resources.

In summary, existing research has achieved notable progress in data annotation, pixel-level reconstruction, parametric representations, multi-resolution mesh modeling, and temporal consistency. Nonetheless, several challenges remain unresolved: the trade-off between detail recovery and temporal smoothness in monocular settings; the absence of a unified, generalizable paradigm for disentangling style and content; the limited capability of unsupervised or weakly supervised approaches to discover styles and generalize across domains; and the difficulty of injecting style in a manner that is both robust and semantically faithful. To address these challenges, this study introduces a spatiotemporal attention-based representation framework combined with a contrastive loss design, aimed at improving both semantic fidelity and style separability. Systematic experiments are conducted in subsequent sections to validate its effectiveness. Furthermore, as motion style remains an inherently abstract concept, it cannot yet be captured with precise linguistic descriptors. To ensure more vivid and realistic stylization, real motion sequences are adopted as style inputs, from which latent style features are extracted to guide the generation of motion that better aligns with real-world patterns.

## 3. Methodology

**Fig 2:**
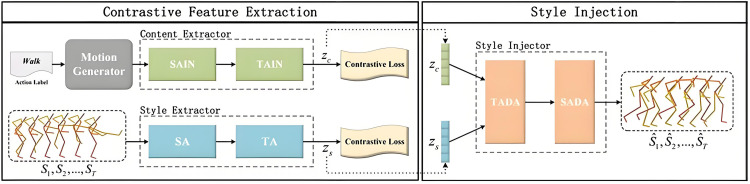
The illustration of Contrastive learning-driven spatiotemporal dynamically adaptive framework.

The overall architecture is illustrated in Fig 2 and comprises two main branches. The first branch generates motion sequences based on action labels using a motion generator, while the second branch is responsible for injecting style into the motion. The style injection process is divided into two stages: style extraction and style injection. In the style extraction stage, motion content features and style features are separately extracted. A style extractor is employed to encode real motion sequences and capture both local and global stylistic attributes. Simultaneously, a content extractor processes motion fragments generated from action labels to extract corresponding content features. The extracted style and content features are subsequently fused through a style injector, and then decoded into a final stylized 3D motion sequence.

The style extractor encodes both local and global patterns from real human motion sequences using a SA module and a TA module. The SA module captures intra-frame spatial relationships among joints, while the TA module models inter-frame temporal dynamics. This process is formally described by Eq. (1), where $W_{SA}$ and $W_{TA}$ denote the parameters of the SA and TA modules, respectively.


$$SF=TA\left(SA\left(S_{1:T};W_{SA}\right),W_{TA}\right)$$ (1)

The content extractor utilizes a SAIN module and a TAIN module to extract motion content features *CF* from the generated sequence ${\hat{P}}_{1:T}$. To ensure that the final stylized sequence exhibits style solely from the target style, any residual stylistic information in ${\hat{P}}_{1:T}$ is removed during content extraction. This operation is represented by Eq. (2), where $W_{SAIN}$ and $W_{TAIN}$ are the corresponding parameters for the SAIN and TAIN modules.


$$CF=TAIN\left(SAIN\left({\hat{P}}_{1:T};W_{SAIN}\right),W_{TAIN}\right)$$ (2)

Next, the style injector incorporates the extracted motion style features *SF* into the content features *CF* via a TADA module and a SADA module. The resulting output sequence ${\hat{S}}_{1:T}=\left\{{\hat{S}}_{1},{\hat{S}}_{2},\cdots ,{\hat{S}}_{T}\right\}$ exhibits the target style ${\hat{S}}_{1:T}$ while accurately executing the action content specified by label *a*. This process is described by Eq. (3), with $W_{SADA}$ and $W_{TADA}$ denoting the parameters of the SADA and TADA modules, respectively.


$${\hat{S}}_{1:T}=SADA\left(TADA\left(SF,CF;W_{TADA}\right),W_{SADA}\right)$$ (3)

### 3.1. Spatial attention module

**Fig 3:**
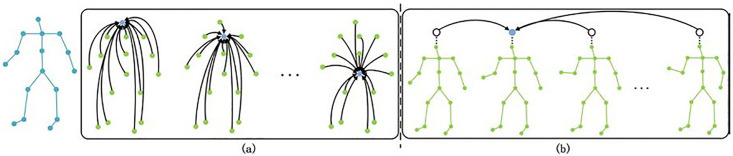
(a) Structure of the SA module; (b) Structure of the TA module.


The SA module applies a multi-head self-attention mechanism within each frame to capture spatial relationships between body joints, as illustrated in Fig 3(a). At time frame *t*, each joint $i^{t}$ is encoded through a learnable linear layer to generate query $q_{i}^{t}$, key $k_{i}^{t}$, and value vectors $v_{i}^{t}$. The similarity between joints $\left(i^{t},j^{t}\right)$ is computed via dot-product between query $q_{i}^{t}$ and key $k_{i}^{t}$, yielding attention weights $\alpha_{ij}^{t}$. These weights are used to compute a weighted sum over the value vectors $v_{j}^{t}$, resulting in an updated embedding vector $z_{i}^{t}$ for joint $i^{t}$. This process is formally expressed in Eq. (4).

$$\alpha_{ij}^{t}=q_{i}^{t}\cdot k_{j}^{t^{⊺}},\forall t\in T,z_{i}^{t}=\sum {\mathrm{\backslash nolimits}}_{j}\text{softmax}\left(\frac{\alpha_{ij}^{t}}{\sqrt{d_{k}}}\right)v_{j}^{t}$$ (4)

### 3.2 Temporal attention module

The TA module conducts an overall study on the dynamic changes of human posture in the time dimension, and the process is shown in Fig 3. The human posture of each frame is regarded as independent, and the correlation between frames is calculated by comparing the characteristic changes of human posture in the time dimension. This process can be expressed as Eq. (5):


$$\alpha_{ij}=q_{i}\cdot k_{j},z_{i}=\sum {\mathrm{\backslash nolimits}}_{j}softmax\left(\frac{\alpha_{ij}}{\sqrt{d_{k}}}\right)v_{j}$$ (5)

Each frame in a motion sequence is considered as a single one. *i* and *j* represent separate time steps, namely the *i*_th_ frame and the *j*_th_ frame. $\alpha_{ij}$ represents the calculated attention weight, which reflects the correlation between the query vector and the key vector. $z_{i}$ represents the overall representation of the features of all posture frames weighted by the attention weights for time step *i*.

### 3.3. Style extractor based on contrastive learning

**Fig 4:**
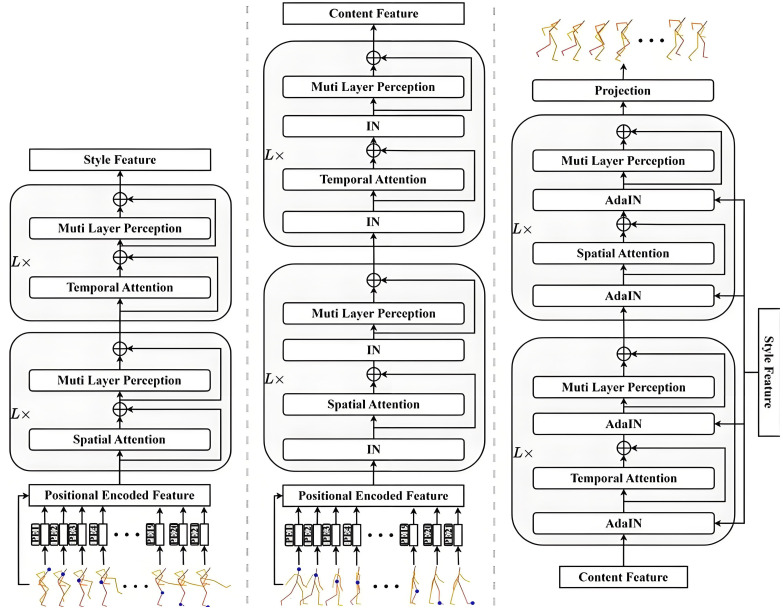
Diagram of the three module components. (a) Style extractor (b) Content extractor (c) Style Injector.


Diagram of the three module components is illustrated in Fig 4. Given a real 3D human motion sequence $S_{1:T}=\left\{S_{1},S_{2},\cdots ,S_{T}\right\}$ as the target style input, the sequence is first fed into the style extractor. Here, $S_{1:T}\in {\mathbb{R}}^{T\times J\times 3}$ and *T* denote the number of frames, and *J* represents the total number of joints in the skeleton. Initially, all joints are mapped into a latent space of dimension *D* using a linear layer, enabling the extraction and learning of high-level joint representations. This process transforms the original positional data into a more expressive feature space. Subsequently, learnable positional embeddings are added to capture spatial dependencies among joints, producing embedded features $E\in {\mathbb{R}}^{T\times J\times D}$. The transformation can be expressed as:

$$E=LN\left(S_{1:T},W_{LN_{1}}\right)+E_{spos_{1}}$$ (6)

where $W_{LN_{1}}$ is the linear transformation and $E_{spos_{1}}$ represents the positional embedding.

The embedded tensor *E* is then input into the SA module, which consists of a spatial self-attention block and a MLP composed of a linear layer and a GELU activation. The spatial attention is first computed to capture local dependencies among joints, followed by a residual connection that mitigates vanishing gradients and accelerates convergence, producing output $SP_{SA}\_out$. The output $SP_{SA}\_out$ is then passed through the MLP, followed by another residual connection to yield output SA_out, formulated as:


$$\begin{array}{c} SP_{SA}\_out=SpatialAttention\left(E,W_{SP_{SA}}\right)+E\\ SA\_out=MLP\left(SP_{SA}\_out,W_{MLP_{SA}}\right)+SP_{SA}\_out \end{array}$$ (7)

where $W_{SP_{SA}}$ and $W_{MLP_{SA}}$ denote the parameters of the SA and MLP blocks, respectively.

The output SA_out is then processed by the TA module, which similarly involves two stages: temporal attention and MLP. Prior to these steps, each frame of the SA module’s output SA_out is treated as a separate timestep and added with positional embeddings to facilitate temporal modeling. This produces features *E*_2_, given by:


$$E_{2}=SA\_out+E_{tpos_{1}}$$ (8)

Temporal multi-head self-attention is then applied to capture dependencies across timesteps, followed by a residual connection producing output $TP_{TA}\_out$. This is passed into an MLP of identical structure, and another residual connection is added to produce output TA_out:


$$\begin{array}{c} TP_{TA}\_out=TemproalAttention\left(E_{2},W_{TP_{TA}}\right)+E_{2}\\ TA\_out=MLP\left(TP_{TA}\_out,W_{MLP_{TA}}\right)+TP_{TA}\_out \end{array}$$ (9)

where $W_{TP_{TA}}$ and $W_{MLP_{TA}}$ denote the parameters of the temporal attention and MLP components, respectively.

The proposed style extractor comprises L stacked SA and TA modules. The extracted style feature *SF* from the source style sequence $S_{1:T}$ is formulated as:


$$SF=TA\left(SA\left(LN\left(S_{1:T},W_{LN_{1}}\right)+E_{spos_{1}},W_{SA}\right)+E_{tpos_{1}},W_{TA}\right)$$ (10)

To enhance the extractor’s style discrimination and generalization capabilities, contrastive learning is introduced during training. This approach minimizes intra-class distances and maximizes inter-class separability in the style feature space. Given a batch of motion sequences $S=\left\{s_{i}|i\in \beta \right\}$ with index set *β* = {1,2,..., *b*_s_}, where *b*_s_ is the batch size, each style feature in the batch is associated with a style label. The contrastive loss is defined as:


Style_conloss=∑\nolimitsi∈β−1|Same(i)|∑\nolimitss∈Same(i)logexp(si:ss/ssτ\nulldelimiterspaceτ)∑\nolimitsa∈A(i)exp(si:ss/ssτ\nulldelimiterspaceτ) (11)

where Same(i) denotes the index set of features with the same style label as $s_{i}$, *A(i)* denotes all other indices in the batch except *i*, and τ is a temperature parameter controlling the sharpness of the similarity distribution.

### 3.4. Content extractor based on contrastive learning

Although the generated motion sequence ${\hat{P}}_{1:T}$, conditioned on action category labels, already contains vivid human motion, it still may contain implicit style information. Therefore, to obtain pure action content representations, the existing style must be removed. Initially, the input motion sequence is mapped to a high-dimensional latent space via a linear layer. The resulting representations are then fed into the SAIN module to extract spatial content features. Before this, positional embeddings $E_{spos_{2}}$ are added to encode spatial relationships among joints, producing embedded features *E*′ as:


$$E^{\prime }=LN\left({\hat{P}}_{1:T},W_{LN_{2}}\right)+E_{spos_{2}}$$ (12)

where $W_{LN_{2}}$ denotes the parameters of the linear layer.

The SAIN module largely mirrors the structure of the SA module used in the style extractor, consisting of a spatial attention block, MLP, and residual connections. However, to explicitly eliminate potential style bias, an instance normalization layer is introduced, which normalizes each sample independently to suppress stylistic variations. The output SAIN_out of the SAIN module is computed as:


$$\begin{array}{c} IN_{SAIN_{1}}\_out=IN\left(E^{\prime },W_{IN_{SAIN_{1}}}\right)\\ SP_{SAIN}\_out=SpatialAttention\left(IN_{SAIN_{1}}\_out,W_{SP_{SAIN}}\right)+IN_{SAIN_{1}}\_out\\ IN_{SAIN_{2}}\_out=IN\left(SP_{SAIN}\_out,W_{IN_{SAIN_{2}}}\right)\\ SAIN\_out=MLP\left(IN_{SAIN_{2}}\_out,W_{MLP_{SAIN}}\right)+IN_{SAIN_{2}}\_out \end{array}$$ (13)

where $W_{SP_{SAIN}}$ and $W_{MLP_{SAIN}}$ represent the spatial attention and MLP parameters, and $W_{IN_{SAIN_{1}}}$, $W_{IN_{SAIN_{2}}}$ denote the parameters of the instance normalization layers.


$$\begin{array}{c} IN_{SAIN_{1}}\_out=IN\left(E^{\prime },W_{IN_{SAIN_{1}}}\right)\\ SP_{SAIN}\_out=SpatialAttention\left(IN_{SAIN_{1}}\_out,W_{SP_{SAIN}}\right)+IN_{SAIN_{1}}\_out\\ IN_{SAIN_{2}}\_out=IN\left(SP_{SAIN}\_out,W_{IN_{SAIN_{2}}}\right)\\ SAIN\_out=MLP\left(IN_{SAIN_{2}}\_out,W_{MLP_{SAIN}}\right)+IN_{SAIN_{2}}\_out \end{array}$$ (13)

The output SAIN_out is then input into the TAIN module. Each frame is first augmented with temporal positional embeddings, followed by instance normalization to ensure consistent distributions across time. Temporal attention is applied to model frame-wise dependencies, and a residual connection is added to produce $TP_{TAIN}\_out$. The final content representation HHH is obtained after passing $TP_{TAIN}\_out$ through the MLP and another residual connection:


$$\begin{array}{c} E_{2}^{\prime }=SAIN\_out+E_{tpos_{2}}\\ IN_{TAIN_{1}}\_out=IN\left(E_{2}^{\prime },W_{IN_{TAIN_{1}}}\right)\\ TP_{TAIN}\_out=TemporalAttention\left(IN_{TAIN_{1}}\_out,W_{TP_{TAIN}}\right)+IN_{TAIN_{1}}\_out\\ IN_{TAIN_{2}}\_out=IN\left(TP_{TAIN}\_out,W_{IN_{TAIN_{2}}}\right)\\ TAIN\_out=MLP\left(IN_{TAIN_{2}}\_out,W_{MLP_{TAIN_{2}}}\right)+IN_{TAIN_{2}}\_out \end{array}$$ (14)

where $W_{IN_{TAIN_{1}}}$ and $W_{IN_{TAIN_{2}}}$ represent the parameters of the instance normalization layers, and $W_{TP_{TAIN}}$, $W_{MLP_{TAIN}}$ are the parameters of the temporal attention and MLP components. The content extractor consists of L stacked SAIN and TAIN modules. It extracts the action content representation from the generated motion ${\hat{P}}_{1:T}$ while discarding residual style features from ${\hat{P}}_{1:T}$, expressed as:


$$CF=TAIN\left(SAIN\left(LN\left({\hat{P}}_{1:T},W_{LN_{2}}\right)+E_{spos_{2}},W_{SAIN}\right)+E_{tpos_{2}},W_{TAIN}\right)$$ (15)

To further accelerate convergence and improve content representation learning, contrastive learning is also employed in training the content extractor. Let 𝐶={𝐶𝑖|𝑖 ∈{1,..., 𝑏s}} be the set of extracted content features for a batch of size *b*_s_, each associated with an action class label. The content contrastive loss is defined as:


Content_conloss=∑\nolimitsi∈{1,2,…,bs}−1|P(i)|∑\nolimitsp∈P(i)logexp(ci·cp/cpτ\nulldelimiterspaceτ)∑\nolimitsb∈B(i)exp(ci·cb/cbτ\nulldelimiterspaceτ) (16)

where 𝑃(𝑖) is the set of indices with the same action label as $c_{i}$, B(i) is the set of all others, and 𝜏 is the temperature parameter.

### 3.5. Style injector

The style injector primarily consists of the TADA and SADA modules, designed to inject the style features 𝑆𝐹 extracted by the style encoder into the motion content features 𝐶𝐹 extracted by the content encoder, thereby generating stylized 3D human motion sequences ${\hat{S}}_{1:T}$. The style features 𝑆𝐹 and motion content features 𝐶𝐹 are first fed into the TADA module, where the style injection is performed through an adaptive instance normalization module. Within this module, an instance normalization layer is initially applied to normalize 𝐶𝐹 along the channel dimension, aiming to reduce redundancy in the feature representation. Subsequently, a linear layer maps the channel dimensions of 𝑆𝐹 to align with those of 𝐶𝐹, facilitating the subsequent style injection. The output of this mapping is denoted as ℎ, which is then split into 𝛾 and 𝛽. The normalized motion features are scaled by 𝛾 and shifted by 𝛽, followed by batch normalization using the mean and variance of each instance to achieve motion style injection, yielding the output Ada_out.This process can be mathematically represented as follows, where $W_{LN}$ and $W_{IN}$ denote the parameters of the linear and instance normalization layers, respectively:


$$\begin{array}{c} h=LN\left(SF,W_{LN}\right)\\ \left[\gamma ,\beta \right]=Chunk\left(h,chunks=2\right)\\ Ada\_out=\gamma \cdot \left(IN\left(CF,W_{IN}\right)\right)+\beta \end{array}$$ (17)

Then, the output of the adaptive instance normalization module Ada_out is passed through a temporal attention module and an MLP, allowing for the weighted fusion of motion content features across different time steps. The functionality of TADA is formally expressed as:


$$\begin{array}{c} Ada_{TADA_{1}}\_out=Adain\left(CF,SF,W_{ADAIN_{TADA_{1}}}\right)\\ TP_{TADA}\_out=TemporalAttention\left(Ada_{TADA_{1}}\_out,W_{TP_{TADA}}\right)+Ada_{TADA_{1}}\_out\\ Ada_{TADA_{2}}\_out=Adain\left(TP_{TADA}\_out,SF,W_{ADAIN_{TADA_{2}}}\right)\\ TADA\_out=MLP\left(Ada_{TADA_{2}}\_out,W_{MLP_{TADA}}\right)+Ada_{TADA_{2}}\_out \end{array}$$ (18)

where $W_{TP_{TADA}}$ denotes the parameters of the temporal attention module in TADA, $W_{ADAIN_{TADA_{1}}}$ and $W_{ADAIN_{TADA_{2}}}$ correspond to the parameters of the adaptive instance normalization module, and $W_{MLP_{TDADA}}$ denotes the parameters of the MLP within TADA.

Finally, the outputs TADA_out and the style features *SF* are input into the SADA module, where style injection is again conducted using an adaptive instance normalization module.


$$\begin{array}{c} Ada\_out=\gamma \cdot \left(IN\left(CF,W_{IN}\right)\right)+\beta \\ Ada_{SADA_{1}}\_out=Adain\left(TADA\_out,SF,W_{ADAIN_{SADA_{1}}}\right)\\ SP_{SADA}\_out=SpatialAttention\left(Ada_{SADA_{1}}\_out,W_{SP_{SADA}}\right)+Ada_{SADA_{1}}\_out\\ Ada_{SADA_{2}}\_out=Adain\left(SP_{SADA}\_out,SF,W_{ADAIN_{SADA_{2}}}\right)\\ SADA\_out=MLP\left(Ada_{SADA_{2}}\_out,W_{MLP_{SADA}}\right)+Ada_{SADA_{2}}\_out \end{array}$$ (19)

where $W_{SP_{SADA}}$ represents the parameters of the spatial attention module in SADA, $W_{ADAIN_{SADA_{1}}}$ and $W_{ADAIN_{SADA_{2}}}$ refer to those of the adaptive instance normalization module, and $W_{MLP_{SADA}}$ indicates the parameters of the MLP.

The final output SADA_out of the SADA module is passed through a linear layer to transform the high-dimensional style-injected motion features into 3D positional coordinates representing the human motion sequence ${\hat{S}}_{1:T}$.


$${\hat{S}}_{1:T}=LN\left(SADA\_out,W_{LN_{3}}\right)$$ (20)

where $W_{LN_{3}}$ denotes the parameters of the linear layer, and the process can be expressed by Eq. (20).

## 4. Experiments and analysis

### 4.1. Datasets and evaluation metrics

#### 4.1.1. Dataset.

The Xia dataset [24] was used for model training in this study. It is a widely adopted motion capture dataset for human motion style analysis. The dataset includes six types of actions—walking, running, jumping, kicking, punching, and action transitions—and eight motion styles: neutral, proud, angry, depressed, confident, childish, elderly, and sexy. It contains approximately 11 minutes of motion clips captured using a Vicon optical motion capture system at a frequency of 120 Hz, resulting in about 79,000 individual frames. For all experiments conducted in this work, the dataset was divided into a training set (85%) and a validation set (15%).

#### 4.1.2 Evaluation metrics.

**Fig 5:**
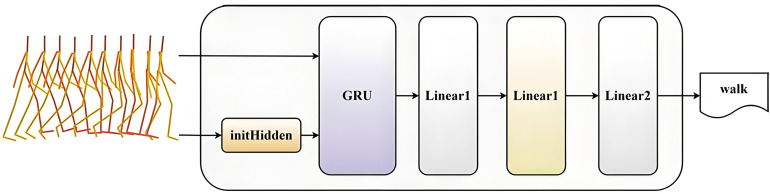
Architecture of the RNN-based action classifier.

To ensure fair and consistent comparison, four commonly used evaluation metrics were employed to assess the generated motion sequences: Fréchet Inception Distance (FID), Accuracy, Diversity, and Multimodality. An RNN-based motion classifier was trained on the Xia dataset [24] to extract motion features and evaluate classification accuracy. The classifier architecture, illustrated in Fig 5, consists of a GRU followed by two fully connected linear layers. The input to the classifier is a human motion sequence, while the output represents the corresponding action label. The GRU serves as a recurrent control unit designed to capture the temporal dependencies within motion sequences. To initialize the hidden state prior to GRU processing, an initHidden method was invoked to provide an initial state, which is subsequently updated according to the current posture data. This mechanism allows the model to effectively capture the correlations and dependencies between motion states at adjacent time steps. The two linear layers perform linear transformations on the extracted features for final action classification.

The diversity metric measures the variance of generated actions across all action categories, reflecting the richness and variety of generated motion classes. Specifically, two subsets of equal size $S_{d}$ are randomly sampled from the set of generated motions corresponding to different action labels. Their motion feature vector sets, denoted as $\left\{v_{1},\ldots ,v_{S_{d}}\right\}$ and $\left\{v_{1}^{\prime },\ldots ,v_{S_{d}}^{\prime }\right\}$, are then extracted. The diversity score is computed as shown in Equation (21).


$$Diversity=\frac{\text{1}}{S_{d}}\sum {\mathrm{\backslash nolimits}}_{i=1}^{S_{d}}{\left\|v_{i}-v_{i}^{\prime }\right\|}_{2}$$ (21)

The multimodality metric evaluates the variation among generated motion sequences conditioned on the same action label, thereby quantifying the model’s ability to produce diverse motions within a single category. Given a set of generated motion sequences containing Caction categories, two subsets of size $S_{l}$ are randomly selected from those belonging to the same action $c_{th}$, and their corresponding feature vector sets $\left\{v_{c,1},\ldots v_{c,S_{l}}\right\}$ and $\left\{v_{c,1}^{\prime },\ldots v_{c,S_{l}}^{\prime }\right\}$ are obtained. The multimodality is calculated as described in Equation (22).


$$Multimodality=\frac{\text{1}}{C\times S_{l}}\sum {\mathrm{\backslash nolimits}}_{c=1}^{C}\sum {\mathrm{\backslash nolimits}}_{i=1}^{S_{l}}{\left\|v_{c,i}-v_{c,i}^{\prime }\right\|}_{2}$$ (22)

Since subjective evaluation methods for assessing motion style quality often vary among individuals and lack reproducibility, this study adopts a quantitative evaluation approach. Following the protocol in [32], Content Recognition Accuracy (CRA) and Style Recognition Accuracy (SRA) are employed to objectively assess the quality of the generated stylized motion sequences. For fair comparison, the same recognition network as in [32] is used to identify both the action and style categories of the generated motion clips. This recognition model is based on the Spatial-Temporal Graph Convolutional Network (ST-GCN), which is specifically designed for human action recognition tasks, taking human motion clips as input and outputting their corresponding action categories. The ST-GCN classifier is trained on the preprocessed Xia dataset, using motion data and corresponding action labels to learn its capability for action-type recognition, which is later used for computing CRA. Similarly, the classifier is trained with motion data and style labels to evaluate its performance in recognizing motion styles, thereby enabling the computation of SRA.

#### 4.1.3. Data preprocessing.

To simplify the dataset, the Xia motion capture data were restructured into a skeleton containing 21 joints, aligning its topology with that of the CMU motion dataset. The human motion information was extracted from the BVH files provided by the Xia dataset. The BVH (Biovision Hierarchy) format, commonly used to describe 3D animation, consists of two main sections: HIERARCHY, which defines the skeletal structure, and MOTION, which records frame-wise motion data. In the HIERARCHY section, the skeleton is defined through a forward kinematic chain starting from the “ROOT” joint, followed by hierarchically connected “JOINT” nodes that represent the limbs and torso. This section encodes the parent–child relationships among joints, their names, offset vectors relative to their parents, rotational Euler angles, and the initial position of the root joint. The MOTION section specifies the number of frames, frame rate, and per-frame motion data, including the position of the root joint and the rotation of all joints.

From each BVH file, the root joint positions, joint rotation data (in Euler angles), joint offsets, and joint names were extracted. The Euler angles were then converted to quaternions to avoid singularities and improve numerical stability. The conversion began by defining the rotation order as “xyz” and normalizing the three corresponding rotation axes. For each frame, the rotation angles around the x-, y-, and z-axes were read as $x_{angle}$, $y_{angle}$, and $z_{angle}$, respectively. Their sine and cosine values were computed, with the sine values multiplied by the respective axis vectors to form the imaginary components and the cosine values forming the real components, thus producing quaternion-based joint rotations.

The global positions of all joints were computed using forward kinematics. First, the local quaternions of each joint were recursively multiplied by their parent’s global quaternion to obtain the global quaternion for each joint. Then, starting from the joints directly connected to the root, each joint’s global position was determined by adding the parent’s global position to its local offset, followed by rotation using the joint’s quaternion.

**Fig 6:**
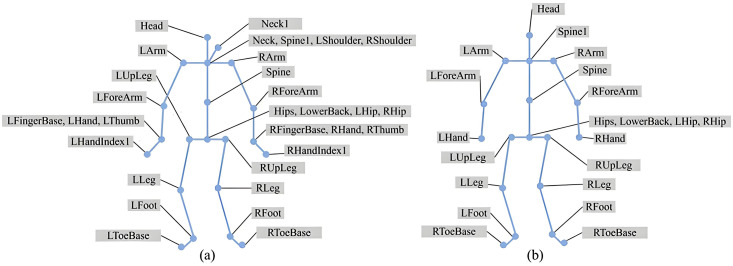
Comparison of the skeletal structures before and after preprocessing: (a) the original 31-joint skeleton from the Xia dataset; (b) the simplified 21-joint skeleton used in this study.

The BVH files in the Xia dataset contain skeletons with 31 joints. However, some joints exhibit zero offsets relative to their parent joints, meaning they overlap spatially and contribute no effective movement. To reduce redundancy and computational overhead, a 21-joint skeleton was selected based on the CMU skeletal standard. Fig 6(a) illustrates the original 31-joint skeleton, and Fig 6(b) shows the simplified 21-joint structure used in this study. This preprocessing effectively reduces the complexity and redundancy of the skeletal data, thereby improving computational efficiency and saving processing time. Finally, the 3D positional coordinates of all joints were stored in .npy format for subsequent model training.

### 4.2. Implementation details

During training, all experiments are conducted under a Windows environment using the PyTorch deep learning framework, with PyCharm as the integrated development environment. An NVIDIA Quadro RTX 5000 GPU is used for model training. In our model, the number of style-injection blocks L is set to 2. The temperature parameter *τ* for both style and content contrastive losses is set to 0.8. The feature space dimensionality is set to 64, with a fixed learning rate of 1e-3 and a batch size of 20.

### 4.3. Comparative analysis

The Xia dataset [24] is utilized to evaluate the model’s capability for 3D human motion generation and style injection. The performance is compared with several state-of-the-art methods. As shown in **Table 1**, the proposed method outperformed others in terms of FID, diversity, and accuracy, indicating strong performance in motion generation.

**Table 1:** Comparison with recent works in motion generation on the Xia dataset.

Method	FID↓	Accuracy↑	Diversity→	Multimodality→
Real	0.01 ± 0.00	96.70 ± 0.09	5.70 ± 0.14	1.01 ± 0.05
Action2Motion [33]	2.49 ± 0.01	61.50 ± 0.03	5.56 ± 0.05	2.32 ± 0.6
ACTOR [34]	0.27 ± 0.00	89.90 ± 2.07	5.56 ± 0.07	1.95 ± 0.12
ASMNet [35]	0.17 ± 0.00	96.60 ± 0.71	5.52 ± 0.08	0.97 ± 0.04
Ours	0.06 ± 0.00	96.70 ± 0.57	5.67 ± 0.16	0.97 ± 0.02

Moreover, to evaluate the quality of the stylized 3D human motion data generated by the proposed model, a comparison was conducted with several state-of-the-art methods specifically designed for human motion style transfer. The results are summarized in **Table 2**. In the table, Real denotes metrics computed from real motion data, * indicates results reproduced in this study, and † represents the results reported in [37]. A lower FID value indicates better alignment between the two distributions. The proposed method achieved an FID of 0.06, which is close to the real data value (0.01) and significantly lower than those of other methods. This suggests that the generated samples more closely approximate the true distribution in both visual and statistical characteristics, thereby reducing distributional shifts and mitigating perceptual and semantic discrepancies. Accuracy and CRA were employed to assess the semantic fidelity and expressive capability of the generated motion sequences, respectively. The proposed method achieved scores of 96.70 and 94.11, which are comparable to the real data, indicating that the generated motions were correctly classified by the action recognizer in both action category and expressive performance, thus preserving semantic consistency. Diversity and Multimodality metrics were used to evaluate the variability and multimodal structure of generated samples under the same action conditions. The proposed method achieved a diversity score close to that of real data, demonstrating its ability to produce reasonable motion variations while maintaining semantic consistency. Moreover, its multimodality value was also comparable to that of real samples, indicating that the generated motions exhibited no excessive mode collapse or unnatural over-segmentation of stylistic modes. SRA designed to assess style transfer effectiveness, reached 89.41, close to the real data score (90.24) and markedly higher than most baselines. This result demonstrates that the proposed style injection mechanism effectively enhances the discriminability of style representations without compromising motion semantics. Overall, the experimental results confirm that the improved style injection branch enables the model to more accurately capture and express distinctive motion and style characteristics. The generated motions exhibit higher expressiveness and discriminability, indicating that the refined model can better learn and represent features across different motion types and styles.

**Table 2:** Comparison with state-of-the-art methods for motion style transfer on the Xia dataset.

Method	CRA↑	SRA↑
Real	96.04 ± 0.00	90.24 ± 0.00
Real*	96.51 ± 0.00	90.69 ± 0.00
Amberman [36] ([37]†)	29.09 ± 1.29	41.97 ± 2.01
Holden [38] ([37]†)	38.93 ± 2.09	41.92 ± 1.77
Motion Puzzle [37]	29.83 ± 1.35	54.94 ± 2.09
ASMNet [35]	74.12 ± 0.00	52.94 ± 0.00
Ours	94.11 ± 0.00	89.41 ± 0.00

Given the potential inequality of variances among methods and the relatively small sample size, a two-sided Welch’s t-test was employed to compare the proposed method (Ours) with each baseline. In addition, Cohen’s d was calculated to quantify the effect size of observed differences. To control the Type I error rate resulting from multiple comparisons, Bonferroni correction was applied separately to the metrics in Table 1 (three baselines) and Table 2 (four baselines). The adjusted significance thresholds were set to α = 0.05/3 ≈ 0.0167 for Table 1 and α = 0.05/4 = 0.0125 for Table 2. Results with p values below the corrected thresholds were marked as statistically significant (p < α). Tables 3 and 4 summarize the detailed statistical test results between Ours and each baseline, including t-values, degrees of freedom (df), unrounded p-values, and corresponding Cohen’s d effect sizes. The overall findings are as follows:

**Table 3:** Statistical test results between the proposed method and each baseline in Table 1.

Baseline vs Ours	Metric	t	df	p	Cohen’s d	Significant after correction
Action2Motion vs Ours	FID	543.34	4.001	6.86e-11	343.64	Yes (p < 0.0167)
	Accuracy	137.90	4.022	1.52e-08	87.21	Yes
	Diversity	1.47	4.774	0.205	0.93	No
	Multimodality	−5.03	4.009	0.00730	−3.18	Yes
ACTOR vs Ours	FID	−95.69	4.222	3.74e-05	11.39	Yes
	Accuracy	5.18	4.103	3.79e-03	2.29	Yes
	Diversity	1.60	4.257	0.180	1.01	No
	Multimodality	−6.20	4.006	3.74e-05	−3.79	Yes
ASMNet vs Ours	FID	−1739.25	8.000	1.34e-23	−1100.00	Yes
	Accuracy	0.25	7.643	0.812	0.16	No
	Diversity	1.88	5.882	0.111	1.19	No
	Multimodality	0.00	5.882	1.000	0.00	No

**Table 4:** Statistical test results between the proposed method and each baseline in Table 2.

Baseline vs Ours	Metric	t	df	p	Cohen’s d	Significant after correction
Amberman vs Ours	CRA	112.70	4.000	3.72e-08	71.28	Yes (p ≪ 0.0125)
	SRA	52.78	4.000	7.72e-07	33.38	Yes
Holden vs Ours	CRA	59.04	4.000	4.93e-07	37.34	Yes
	SRA	59.99	4.000	4.62e-07	37.94	Yes
MotionPuzzle vs Ours	CRA	106.47	4.000	4.67e-08	67.34	Yes
	SRA	36.88	4.000	3.23e-06	23.32	Yes
ASMNet vs Ours	CRA	3.16e5	8.000	1.12e-41	1.998e5	Yes
	SRA	5.77e5	8.000	9.16e-44	3.647e5	Yes

The proposed method achieved significantly lower FID values than all baseline methods (Bonferroni-corrected p ≪ 0.01), indicating that the distributional gap between generated and real samples was statistically smaller. The proposed method outperformed Action2Motion and ACTOR with statistical significance (corrected p < 0.0167), while showing no significant difference from ASMNet (p > 0.0167). This suggests that Ours achieved an accuracy comparable to the strongest baseline. Differences between Ours and the main baselines did not meet the Bonferroni-corrected significance threshold, implying that no statistically stable improvement or degradation in sample diversity was observed. Significant differences were found between Ours and Action2Motion as well as ACTOR (corrected p < 0.0167), while differences with ASMNet were not significant. For style-related metrics, the proposed method exhibited highly significant improvements over all comparison methods (Bonferroni-corrected p ≪ 0.0125), with extremely large Cohen’s d values. These results strongly support the superior style expressiveness and discriminability achieved by the proposed model.

**Fig 7:**
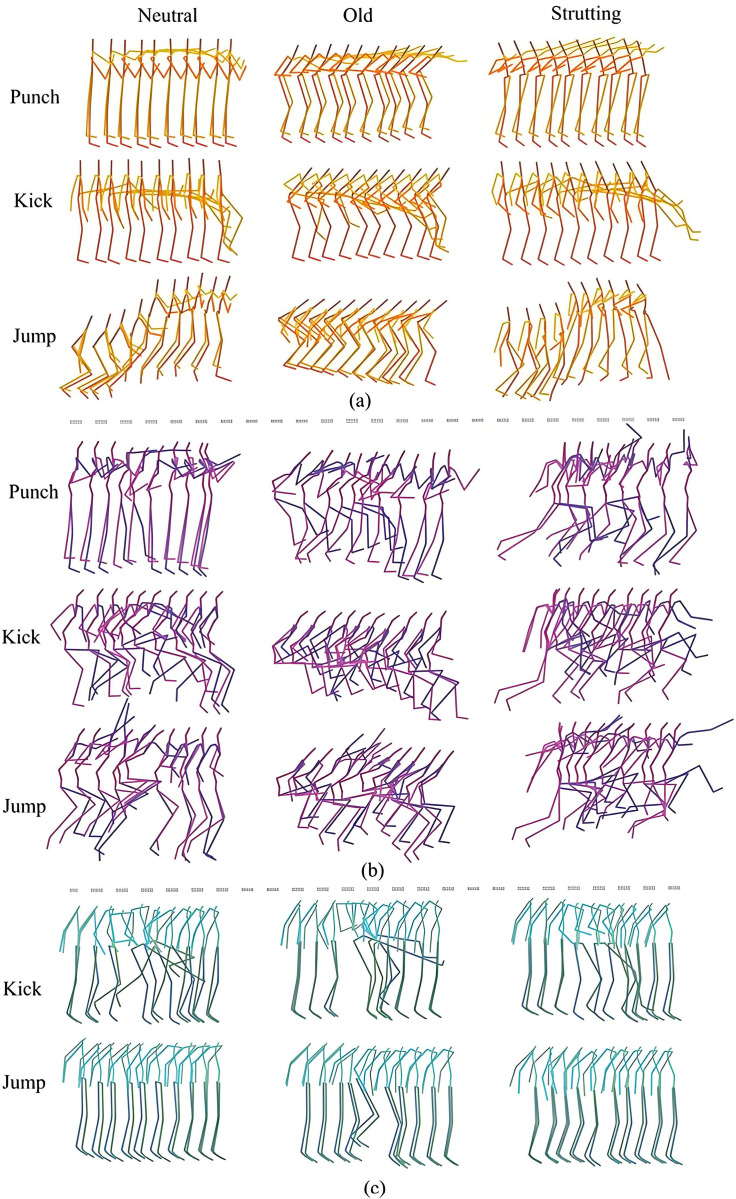
Visual comparison of the proposed method (a), MotionCLIP (b), and MotionDiffuse (c).

Although most existing studies primarily focus on motion generation tasks, a few have recognized the importance of motion style in influencing generation quality. Therefore, visual comparisons are conducted between the sequences generated by the proposed method and those from MotionCLIP and MotionDiffuse, as shown in Fig 7. The orange sequences (Fig 7(a)) correspond to the results from our method, while the purple (Fig 7(b)) and green (Fig 7(c)) sequences represent those from MotionCLIP and MotionDiffuse, respectively. The figure presents visualizations of sequences performing actions such as “punch,” “kick,” and “jump” under three styles: “neutral,” “elderly,” and “upright.” It is evident that MotionDiffuse produces weak stylistic variations across different styles for the same action, failing to clearly represent style features. MotionCLIP shows observable differences in body curvature across styles, with the greatest curvature in the “elderly” style, followed by the “upright” style, and the least in the “neutral” style. However, when viewed in isolation, individual sequences lack clearly recognizable style traits. In contrast, the sequences generated by our method display distinctive characteristics for each style: the “elderly” style exhibits a hunched torso and bent limbs, the “neutral” style reflects standard execution of motion, and the “upright” style features exaggerated backward arm extension. These visual results further validate the effectiveness of the proposed contrastive learning-based method for stylized 3D human motion generation, demonstrating improved style separability. Even for actions like jumping or punching, distinct stylistic attributes are clearly conveyed.

### 4.3. Ablation studies

Extensive ablation studies are conducted on the Xia dataset under controlled conditions to validate the effectiveness of the proposed motion generation model. The model incorporates spatial attention and temporal attention modules for motion style extraction and injection. Based on this, several modules are proposed, including the SA and TA modules, as well as their extensions: SAIN, SADA, TAIN, and TADA. To assess the effectiveness of these modules, models using only temporal attention and only spatial attention are implemented while keeping other experimental settings unchanged. The results, presented in Table 5(a) and 5(b), show that although CRA scores are comparable to real data, the SRA values remained significantly lower than those of the proposed method. This indicates that the combined use of spatial and temporal attention is more effective, enabling a more comprehensive understanding of style features from both global and local perspectives, thereby enhancing the stylistic expressiveness of the generated motion sequences.

**Table 5:** Ablation study results.

Method	CRA↑	SRA↑
Real	96.51 ± 0.00	90.69 ± 0.00
(a) Spatial attention module	85.88 ± 0.00	60.00 ± 0.00
(b) Temporal attention module	94.11 ± 0.00	58.82 ± 0.00
(c) Recon + Content_pre	90.58 ± 0.00	23.52 ± 0.00
(d) Recon + Content_con	95.29 ± 0.00	72.94 ± 0.00
(e) Recon + Style_con	92.94 ± 0.00	75.29 ± 0.00
(f) Recon + Content_pre + Content_con	91.76 ± 0.00	58.83 ± 0.00
(g) Recon + Content_pre + Style_con	95.29 ± 0.00	40.00 ± 0.00
Ours	94.11 ± 0.00	89.41 ± 0.00

Additionally, contrastive learning is introduced for human motion style extraction through the proposed style contrastive loss and content contrastive loss. To validate their effectiveness, experiments are conducted by varying the loss function combinations while keeping other settings fixed. Results are shown in Table 5(c)–3(g), where *Recon* denotes reconstruction loss, *Content_pre* denotes content preservation loss, *Content_con* denotes content contrastive loss, and *Style_con* denotes style contrastive loss.

In Table 5(c), the model employing reconstruction loss and content preservation loss achieved a CRA of 90.58, indicating improved content representation, but the SRA remained low, suggesting poor style expressiveness. In contrast, models in Table 5(d) and 5(e), which employed contrastive learning, achieved both high CRA and SRA scores (SRA > 70), demonstrating that the proposed contrastive losses significantly enhance the accuracy of style feature extraction.

To further validate the importance of contrastive losses for feature extraction, combinations of content preservation with content contrastive loss and style contrastive loss are tested. Results shown in Table 5(f) and 5(g) indicate that while the addition of contrastive loss improved SRA compared to Table 5(c), the values still fell short of those in Table 5(d) and 5(e). Therefore, the content preservation loss is ultimately removed, and only style and content contrastive losses are retained for training. This configuration enabled the model to achieve consistently high CRA and SRA scores, confirming the superiority of the proposed design.

The conclusions drawn from the ablation study on the four types of losses are as follows: Reconstruction loss ensures that the generated sequences preserve the basic motion shapes and temporal consistency, serving as a necessary component for maintaining the baseline CRA performance. However, when used alone, it is insufficient for learning discriminative style representations, as evidenced by the significantly lower SRA observed in Table 5(c) for the SRA metric compared with configurations including contrastive loss. Content preservation loss is designed to explicitly maintain the original content (i.e., motion semantics), this loss prevents excessive distortion of action semantics during style injection. Consequently, when combined with reconstruction loss alone (Table 5(c)), it improves CRA. Nevertheless, experiments indicate that the simultaneous use of content preservation loss and contrastive loss—particularly style contrastive loss—can conflict with the goal of style disentanglement, resulting in decreased style discriminability (Tables 5(f)/(g) show a notable drop in SRA compared with using contrastive loss alone). This suggests that, if improperly weighted or designed, content preservation loss may hinder the separability of style representations while enforcing content consistency. Content contrastive loss by pulling together content representations of the same action across different styles and pushing apart representations of different actions, this loss enhances intra-class aggregation and inter-class separability in the embedding space. When used alongside reconstruction loss (Table 5(d)), it preserves high CRA while positively influencing SRA, likely because clearer content boundaries reduce intra-class noise, allowing the style encoder to focus on residual style differences.

Style contrastive loss directly improves SRA by increasing inter-style distances and reducing intra-style variance (Table 5(e)). When both content contrastive loss and style contrastive loss are applied while omitting content preservation loss, high action semantic consistency (CRA) is maintained alongside optimal style discriminability (SRA). This observation motivates our final design choice to exclude content preservation loss and adopt the dual contrastive loss combination.

## 5. Conclusion

This study proposes a spatiotemporal attention and contrastive learning-based framework for stylized 3D human motion generation. Extensive ablation studies, quantitative evaluations (FID, Accuracy/CRA, SRA, Diversity, Multimodality), and qualitative visualizations demonstrate that the proposed method substantially enhances style discriminability while preserving action semantic fidelity, producing generative distributions closer to real data and achieving overall performance improvements in the stylized 3D motion generation task. Nevertheless, several limitations remain. Lack of subjective evaluation: Large-scale human perceptual studies are absent, leaving the consistency and naturalness of style perception unverified; although automatic metrics and visualizations are consistent, they cannot fully substitute for human judgment. Training overhead and hyperparameter sensitivity: The incorporation of attention modules and contrastive losses incurs high computational cost, and potential conflicts among loss weights necessitate more robust strategies for weight balancing.

Future work will focus on large-scale cross-dataset validation across diverse acquisition devices, action sets, and cultural backgrounds, combined with unsupervised or weakly supervised clustering to automatically discover style subcategories and reduce reliance on manual style annotations. In addition, adaptive loss weighting mechanisms based on dynamic training feedback will be investigated to mitigate conflicts between content preservation and contrastive objectives. Finally, model compression techniques (e.g., pruning, distillation) and efficient attention implementations will be explored to reduce inference latency, enabling deployment in real-time or resource-constrained scenarios.
